# 99. COVID-19 Vaccine related Attitudes and Beliefs among Black and/or Hispanic respondents in Brooklyn, NY: Lessons Learned

**DOI:** 10.1093/ofid/ofac492.177

**Published:** 2022-12-15

**Authors:** Lalitha Parameswaran, Mark Mulligan, Yangjin Park

**Affiliations:** NYU Grossman School of Medicine, New York, New York; NYU Grossman School of Medicine, New York, New York; NYU Silver School of Social Work, New York, New York

## Abstract

**Background:**

Vaccine hesitancy remains a significant barrier against COVID-19 vaccination uptake in Black/Hispanic population. The objectives of our study were to assess attitudes such as trust in the process of vaccine development, its approval and its stakeholders and mistrust due to racial discrimination to determine their relationship to vaccine receipt.

**Methods:**

We collected close-ended survey responses in English/Spanish among participants self-identifying as Black/Hispanic in ambulatory clinics affiliated to NYU Langone Health across Brooklyn in June 2021. The survey consisted of demographic questions, and those assessing components outlined in objectives.

**Results:**

A total of 156 participants answered the survey; majority being women (77%), < 50years old (62%), employed full time (51%), annual household income < $75,000 (75%). A vast majority reported trusting their healthcare providers (91%). Of the respondents, 67% (105) self-reported receiving the COVID-19 vaccination, which was higher than the prevailing rate at that time in this population in Brooklyn. Significant proportions of vaccinated group compared to unvaccinated group reported (Table1): understanding how vaccines worked overall (85% vs 53%); trusted their healthcare providers’ advice to get vaccinated (82% vs 50%); trusted the scientists who created and tested the vaccine (80% vs 48%); agreed that it was very important that the community gets vaccinated (82% vs 27%), and it was people’s responsibility to get vaccinated to stop the spread of virus in their community (96% vs 70%); agreed that confidence in the vaccine’s safety (86% vs 50%) and effectiveness (85% vs 52%) were very important. Unvaccinated group reported concerns about the side effects of vaccines, and greater doubt about adequacy of testing for safety and effectiveness of vaccine in their racial/ethnic group, and mistrust with government.

**Conclusion:**

Despite higher self-reported COVID-19 vaccine uptake rate in Black/Hispanic study respondents in June 2021, significant mistrust and concerns about the vaccine remain. The effect of racial discrimination and mistrust can mitigated by greater trust in healthcare team, and future interventions can test this hypothesis in order to improve vaccine uptake in these populations.

**Disclosures:**

**All Authors**: No reported disclosures.

